# Association Between Social Participation and Instrumental Activities of Daily Living Among Community-Dwelling Older Adults

**DOI:** 10.2188/jea.JE20150253

**Published:** 2016-10-05

**Authors:** Kimiko Tomioka, Norio Kurumatani, Hiroshi Hosoi

**Affiliations:** Nara Prefectural Health Research Center, Nara Medical University, Kashihara, Nara, Japan

**Keywords:** social participation, instrumental activities of daily living, successful aging, functional decline, gender difference, elderly

## Abstract

**Background:**

Population-based data examining the relationship between social participation (SP) and instrumental activities of daily living (IADL) are scarce. This study examined the cross-sectional relationship between SP and IADL in community-dwelling elderly persons.

**Methods:**

Self-administered questionnaires were mailed to 23 710 residents aged ≥65 years in Nara, Japan (response rate: 74.2%). Data from 14 956 respondents (6935 males and 8021 females) without dependency in basic activities of daily living (ADL) were analyzed. The number, type, and frequency of participation in social groups (SGs) were used to measure SP. SGs included volunteer groups, sports groups, hobby groups, senior citizens’ clubs, neighborhood community associations, and cultural groups. IADL was evaluated using the Tokyo Metropolitan Institute of Gerontology Index of Competence. Logistic regression models stratified by gender were used.

**Results:**

After adjustment for putative confounding factors, including demographics, health status, life-style habits, ADL, depression, cognitive function, social networks, social support, and social roles, participation in various SGs among both genders was inversely associated with poor IADL, showing a significant dose-response relationship between an increasing number of SGs and a lower proportion of those with poor IADL (*P* for trend <0.001). A significant inverse association between frequent participation and poor IADL was observed for all types of SGs among females, whereas the association was limited to sports groups and senior citizens’ clubs among males.

**Conclusions:**

Our results show that participation in a variety of SGs is associated with independent IADL among the community-dwelling elderly, regardless of gender. However, the beneficial effects of frequent participation on IADL may be stronger for females than for males.

## INTRODUCTION

Social participation (SP) is considered a key dimension of successful aging.^1^ Previous studies have found that SP is a determinant of many favorable health outcomes, such as longevity,^2^^,^^3^ better physical^4^ or cognitive^5^ performance, and better mental health.^6^ Although these previous studies suggest that SP has a potent influence on older adults’ health, it is also important to explore what influence it may have on an elderly person’s ability to perform tasks necessary to live independently in the community (ie, instrumental activities of daily living [IADL]).^7^ However, few studies of SP in senior citizens have focused on IADL as an outcome.^8^

A previous Japanese prospective cohort study reported that participation in specific types of social groups prevented incident functional disability.^9^ Findings from a nationally representative American sample demonstrated that volunteering in moderate amounts reduced risk of mortality to a greater degree than non-volunteering or high amounts of volunteering.^10^ These studies^9^^,^^10^ suggest that the relationship between SP and IADL may vary depending on the type and frequency of SP. Additionally, some prior studies of community-dwelling elderly have assessed gender differences in the impacts of SP on physical functioning, but those findings are inconsistent. In a Japanese study, social capital, including social participation (ie, belonging to a hobby/interest group) may affect the onset of functional disability for women but not for men.^11^ In contrast, a Danish longitudinal study showed that low SP, including poor visitation^12^^,^^13^ and poor social activities outside the home,^12^ was an independent risk factor of onset of mobility disability among males only.

Some findings have indicated that there are no significant differences between males and females with regard to the association between SP and physical functioning among the elderly.^4^^,^^9^^,^^14^ However, we believe that gender differences in the association between SP and IADL are plausible because males can benefit more from spousal support and females more from other people’s support,^15^ and there are gender differences in years free of IADL disability.^16^ Although one study of community-dwelling elderly reported that SP was associated with a decreased risk of incident disability in IADL,^8^ this study lumped together participation in different types of social activities and did not fully consider gender differences in the association between SP and IADL. Revealing what forms of participation (ie, participation in all or particular types of social groups, and frequent or rare participation) are associated with independence of IADL, and whether the association between SP and IADL differs by gender, can promote a greater understanding of how to achieve active aging.

The purpose of this study was to examine the cross-sectional relationship between IADL and SP according to the type and frequency of participation in social groups with regard to gender among community-dwelling elderly.

## METHODS

### Participants

Data were obtained from the Nara Healthy Life Expectancy Study, a cross-sectional community survey conducted in three urban cities in Nara prefecture, located in the western part of Japan. From March to May 2014, these city offices distributed self-administered postal questionnaires to community-dwelling individuals aged 65 years and older. The survey was conducted using a complete census (complete enumeration) of one city and a random sampling method stratified by region, age, and gender in the other two cities. Figure shows the procedure for selecting subjects. Of the 23 710 mailed questionnaires, 17 591 were returned (response rate: 74.2%). Among them, 1867 subjects with missing values for the questions regarding ADL, IADL, social role, and/or SP were excluded. Additionally, 768 individuals with dependency in basic activities of daily living (ADL), who were identified by using the Barthel Index^17^ (a score of <60), were excluded because basic ADL has an influence on higher-level functional capacity and SP.^18^ In total, 2635 returned questionnaires were excluded, resulting in 14 956 subjects (6935 males and 8021 females) available for the present study.

**Figure.**
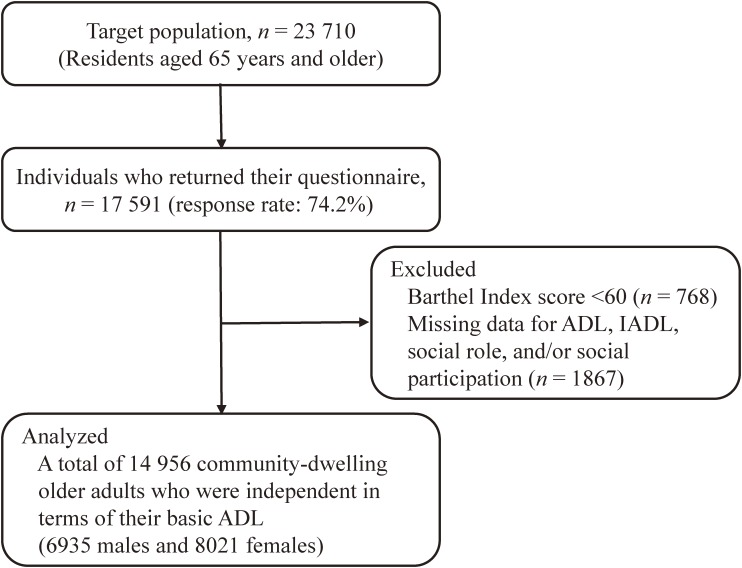
Selection of subjects. ADL, activities of daily living; IADL, instrumental activities of daily living.

All study participants provided signed informed consent. This study protocol was approved by the Nara Medical University Ethics Committee (approval number 990).

### Assessment of instrumental activities of daily living

IADL was evaluated using the 5-item IADL subscale of the Tokyo Metropolitan Institute of Gerontology Index of Competence (TMIG-IC). The TMIG-IC (see eTable 1) was developed to measure the competence required for community-residing elderly to live autonomously,^19^ and its reliability and validity have been confirmed.^20^ The respondents select either “yes” (one point) or “no” (zero point). A full score of five categorized the study subject as independent, and a score of 0–4 categorized the subject as dependent.^21^ Thus, participants were divided into two groups according to IADL score: independent IADL (5 points) and poor IADL (<5 points).

### Assessment of social participation

We defined SP as the person’s engagement in social groups. In line with previous studies,^9^^,^^22^^,^^23^ SP was classified into six types: volunteer groups, sports groups, hobby groups, senior citizens’ clubs, neighborhood community associations, and cultural groups. We asked subjects about their frequency of participation in each group: ≥4 times per week, several times per week, once per week, several times per month, several times per year, or never. Because there were few responses of “≥4 times per week” and “several times per week” (eTable 2), we re-categorized these SP variables into groups of once or more a week, several times per month, several times per year, and non-participation. Additionally, we counted the number of social groups participated in and formed four classifications: 0 groups, 1 group, 2 groups, and ≥3 groups.

### Assessment of activities of daily living

ADL was evaluated using the Barthel Index (score range, 0–100).^17^ A full score of 100 was defined as independent, a score of 60–99 as partially dependent, and a score <60 as dependent (ie, subjects with dependence in basic ADL).

### Covariates

In line with previous studies,^24^^–^^27^ age, family structure, body mass index (BMI), pensions, occupational status, the number of medications used, self-reported medical conditions, self-rated health, smoking status, alcohol consumption, ADL, depression, and cognitive function were used as covariates that may correlate with SP and IADL. Since social relationships, such as social networks, social support, and social roles, may be potential mechanisms for SP to influence the health of the elderly,^18^^,^^28^ these factors were also used as covariates. Information on age and gender was obtained from the municipal offices, and information on other covariates was gleaned from the questionnaire.

Family structure was categorized as living alone, living only with one’s spouse, and other. BMI was subdivided into underweight (<18.5 kg/m^2^), normal (18.5 to <25.0 kg/m^2^), and overweight (≥25.0 kg/m^2^) categories. Pensions indicated socioeconomic status. Pensions categories included national pension, employees’ pension, mutual aid association pension, and other (eg, disability or survivor pension). Occupational status was dichotomized into subjects with or without a job with an income. Medication numbers were labeled as 0 (no medication), 1–2, 3–4, or ≥5 (polypharmacy). Current medical status was evaluated by the question, “Are you now receiving treatment?” to which subjects replied “yes” or “no”. The number of comorbidities (hypertension, stroke, heart diseases, diabetes mellitus, dyslipidemia, chronic respiratory disease, digestive system disease, urogenital disease, musculoskeletal disorder, otological disease, ophthalmologic disease, and cancer) under medical treatment was categorized as 0, 1, 2, 3, or ≥4. Self-rated health was subdivided into very good, good, fair, and poor categories. Smoking categories included never-smoker, former smoker, and current smoker. Alcohol consumption was subdivided into nondrinker, social drinker, occasional drinker, and daily drinker categories. ADL was categorized as 100 full mark (independent) or 60–99 points (poor), based on their Barthel Index scores.^17^ The 5-item short form of Geriatric Depression Scale (GDS-5; score range, 0–5)^29^ was used to determine depression. Participants who had ≥2 points for GDS score were regarded as being depressed. The Cognitive Performance Scale (CPS; score range, 0–6)^30^ was used to assess cognitive function. A score of ≥1 was defined as the presence of poor cognitive functioning. Social network size was defined as the number of children, family, and friends that the participants saw at least once a month^31^ and was categorized as 0, 1–2, 3–5, or ≥6. Social support was defined as the number of children, family, and friends who looked after participants when they were sick and/or listened to their concerns and complaints and was categorized as 0 (no support), 1–2, 3–4, or ≥5.^32^ Social role was categorized as 4 points (independent) or <4 points (poor), based on their TMIG-IC subscale scores.^19^ A category entitled “missing” was used for values that were missing in question responses.

### Statistical analyses

A multiple logistic regression analysis was carried out using “with or without poor IADL (IADL score <5 or 5)” as a dependent variable. The independent variable was SP (“the number of social groups” and “the type and frequency of SP”). The results were shown as odds ratios (ORs) with 95% confidence intervals (CIs). In each model, no social group participation was set as the referent category. In model 1, regression analysis was performed with the simultaneous forced entry of age (continuous), family structure (reference: the living alone group), BMI (reference: the normal group), pensions (reference: the national pension group), occupational status (reference: the subjects without a job), the number of medications used (reference: the none group), self-reported medical conditions (reference: the subjects without a disease), self-rated health (reference: the very good group), smoking (reference: the never-smokers), alcohol intake (reference: the nondrinkers), ADL (reference: the subjects with independent ADL), depression (reference: the subjects without depression), cognitive function (reference: the subjects without poor cognitive functioning) as covariates. In model 2, social networks (reference: the ≥6 group), social support (reference: the ≥5 group), and social roles (reference: the independent group) were added to the variables in model 1. For the association of the number of social groups with poor IADL, we also conducted a linear trend test to assess the dose-response relationship. To examine whether the relationship between SP and poor IADL varied by gender, we performed analyses stratified by gender. The level of significance was 0.05 (two-tailed). Statistical analyses were performed using SPSS (version 17.0; SPSS Japan Inc., Tokyo, Japan).

## RESULTS

### General characteristics of the participants

Among the 14 956 subjects, the prevalence of poor IADL was 19.0% for males and 12.3% for females, showing a significant difference between genders (*P* < 0.001). As shown in eTable 3, males were more likely to live only with their spouse, be employed, be smokers and daily drinkers, and have poor ADL and a low level of social relationships, while females were more likely to be older and underweight, receive a national pension, and have depression. Regarding SP (eTable 2), the proportion of subjects who did not participate in any group was similar between males and females, but males were more likely to participate in volunteer groups, sports groups, and neighborhood community associations than females.

### Comparison between subjects with poor IADL and those with independent IADL

Table 1 shows the characteristics of the study population with and without poor IADL by gender. Regardless of gender, subjects with poor IADL were significantly older; more likely to be underweight, non-working, and polypharmatic; had more chronic diseases; had worse self-rated health; were less likely to be daily drinkers; had poorer ADL; were more likely to be depressed and have poor cognitive function; had fewer social relationships; and had less participation in all types of SP than those without poor IADL. However, the groups did not differ in distribution of smoking status. Because there were no females with poor IADL who participated in neighborhood community associations more than once per week, the frequency of neighborhood community associations was re-categorized as non-participation, several times a year, and once or more a month in the logistic regression analysis.

**Table 1.** Characteristics of the study population with and without poor instrumental activities of daily living by gender (*n* = 14 956)

	Males (*n* = 6935)					Females (*n* = 8021)				
	
	Poor IADL (*n* = 1319)		Independent IADL (*n* = 5616)		*P*^a^	Poor IADL (*n* = 985)		Independent IADL (*n* = 7036)		*P*^a^
	*n*	%	*n*	%		*n*	%	*n*	%	
**Demographics & health status**										
Age ≥75 years	645	48.9	1988	35.4	<0.001	831	84.4	2494	35.4	<0.001
Living with only spouse	680	51.6	2823	50.3	0.409	151	15.3	2636	37.5	<0.001
Underweight (BMI <18.5 kg/m^2^)	101	7.7	254	4.5	<0.001	180	18.3	715	10.2	<0.001
National pension	221	16.8	560	10.0	<0.001	437	44.4	3176	45.1	0.657
Subjects with a job	265	20.1	1633	29.1	<0.001	10	1.0	1031	14.7	<0.001
Subjects with polypharmacy	488	37.0	1281	22.8	<0.001	530	53.8	1436	20.4	<0.001
Number of comorbidities ≥4	175	13.3	507	9.0	<0.001	210	21.3	582	8.3	<0.001
Self-rated health fair or poor	449	34.0	916	16.3	<0.001	517	52.5	1140	16.2	<0.001
**Life-style habits**										
Smoking status: current/former	963	73.0	3983	70.9	0.137	83	8.4	597	8.5	1.000
Alcohol intake: daily drinkers	454	34.4	2402	42.8	<0.001	31	3.1	469	6.7	<0.001
**Physiological and psychological factors**										
Independent ADL	761	57.7	4606	82.0	<0.001	166	16.9	5235	74.4	<0.001
Depression	543	41.2	1098	19.6	<0.001	609	61.8	1663	23.6	<0.001
Poor cognitive function	566	42.9	944	16.8	<0.001	597	60.6	991	14.1	<0.001
**Social relationships**										
Social networks: none	411	31.2	791	14.1	<0.001	376	38.2	613	8.7	<0.001
Social support: <2	759	57.5	3053	54.4	0.039	591	60.0	3375	48.0	<0.001
Social roles: poor	1020	77.3	2815	50.1	<0.001	930	94.4	2626	37.3	<0.001
**Social participation**										
Number of social groups										
0	647	49.1	1538	27.4	<0.001	741	75.2	1922	27.3	<0.001
1	264	20.0	1258	22.4		160	16.2	1550	22.0	
2	174	13.2	1077	19.2		58	5.9	1294	18.4	
≥3	234	17.7	1743	31.0		26	2.6	2270	32.3	
**Type and frequency of social participation**										
Volunteer groups										
Non-participation	1125	85.3	4241	75.5	<0.001	934	94.8	5585	79.4	<0.001
Several times a year	67	5.1	486	8.7		17	1.7	408	5.8	
Several times a month	62	4.7	431	7.7		17	1.7	545	7.7	
Once or more a week	65	4.9	458	8.2		17	1.7	498	7.1	
Sports groups										
Non-participation	1040	78.8	3556	63.3	<0.001	960	97.5	4801	68.2	<0.001
Several times a year	51	3.9	439	7.8		3	0.3	162	2.3	
Several times a month	71	5.4	547	9.7		3	0.3	351	5.0	
Once or more a week	157	11.9	1074	19.1		19	1.9	1722	24.5	
Hobby groups										
Non-participation	961	72.9	3078	54.8	<0.001	900	91.4	3633	51.6	<0.001
Several times a year	104	7.9	650	11.6		22	2.2	410	5.8	
Several times a month	118	8.9	931	16.6		38	3.9	1430	20.3	
Once or more a week	136	10.3	957	17.0		25	2.5	1563	22.2	
Senior citizens’ clubs										
Non-participation	1168	88.6	4769	84.9	<0.001	875	88.8	5791	82.3	<0.001
Several times a year	67	5.1	340	6.1		51	5.2	422	6.0	
Several times a month	55	4.2	312	5.6		47	4.8	562	8.0	
Once or more a week	29	2.2	195	3.5		12	1.2	261	3.7	
Neighborhood community associations										
Non-participation	930	70.5	3092	55.1	<0.001	916	93.0	4162	59.2	<0.001
Several times a year	294	22.3	1833	32.6		61	6.2	2276	32.3	
Several times a month	61	4.6	497	8.8		8	0.8	461	6.6	
Once or more a week	34	2.6	194	3.5		0	0.0	137	1.9	
Cultural groups										
Non-participation	1192	90.4	4673	83.2	<0.001	956	97.1	5342	75.9	<0.001
Several times a year	58	4.4	435	7.7		10	1.0	544	7.7	
Several times a month	43	3.3	327	5.8		13	1.3	653	9.3	
Once or more a week	26	2.0	181	3.2		6	0.6	497	7.1	

ADL, activities of daily living; IADL, instrumental activities of daily living.

^a^Differences between subjects with or without poor IADL were analyzed using Fisher’s exact test.

### Cross-sectional relationship between SP and IADL

Table 2 shows the ORs for poor IADL in association with the number of social groups. In the crude model, participating in ≥1 groups was significantly associated with lower odds of poor IADL compared with non-participation in both genders. After adjusting for covariates (model 1), the association between the number of groups and poor IADL was attenuated but remained statistically significant. Significant dose-response relationships were observed between increasing number of social groups and lower proportion of individuals with poor IADL, regardless of gender (*P* for trend <0.001 in both genders). After additional adjustment for social networks, social support, and social roles (model 2), the ORs tended towards 1.00 but remained basically unchanged from model 1 for both genders: the ORs for poor IADL in males participating in 1 group, 2 groups, and ≥3 groups were 0.74 (95% CI, 0.62–0.89), 0.70 (95% CI, 0.57–0.86), and 0.63 (95% CI, 0.52–0.78), respectively, and the ORs for poor IADL in females participating in 1 group, 2 groups, and ≥3 groups were 0.60 (95% CI, 0.47–0.77), 0.41 (95% CI, 0.29–0.58), and 0.15 (95% CI, 0.09–0.23), respectively, compared to subjects with non-participation in social groups.

**Table 2.** Odds ratios for poor instrumental activities of daily living associated with the number of social groups stratified by gender

	Crude		Model 1^a^		Model 2^b^	
		
	OR	(95% CI)	OR	(95% CI)	OR	(95% CI)
**Males (*n* = 6935)**						
Number of social groups						
0	1.00		1.00		1.00	
1	0.50	(0.43–0.59)	0.68	(0.57–0.81)	0.74	(0.62–0.89)
2	0.38	(0.32–0.46)	0.60	(0.50–0.74)	0.70	(0.57–0.86)
≥3	0.32	(0.27–0.38)	0.49	(0.41–0.59)	0.63	(0.52–0.78)
*P* for trend	<0.001		<0.001		<0.001	
**Females (*n* = 8021)**						
Number of social groups						
0	1.00		1.00		1.00	
1	0.27	(0.22–0.32)	0.47	(0.37–0.59)	0.60	(0.47–0.77)
2	0.12	(0.09–0.15)	0.27	(0.20–0.37)	0.41	(0.29–0.58)
≥3	0.03	(0.02–0.04)	0.08	(0.05–0.13)	0.15	(0.09–0.23)
*P* for trend	<0.001		<0.001		<0.001	

CI, confidence interval; OR, odds ratio.

^a^Adjusted for age, family structure, BMI, pensions, occupational status, the number of medications used, self-reported medical conditions, self-rated health, smoking, alcohol consumption, activities of daily living, depression, and cognitive function.

^b^In addition to the factors in Model 1, social networks, social support, and social roles were included.

Table 3 shows the ORs for poor IADL associated with the type and frequency of SP. In the crude model, only participation in senior citizens’ clubs several times per year was not associated with poor IADL in both genders; after adjusting for covariates (model 1), significant associations were observed. For males, after adjusting for covariates (model 1), all types of SP had protective effects on poor IADL, but frequent participation (ie, ≥once per week) was no longer significant for neighborhood community associations and cultural groups. In contrast, females had a significant inverse association between frequent participation and poor IADL for all types of SP, even in model 1, which adjusted for covariates. Additionally, all types and all frequencies of SP except for participation in sports groups several times per year were significantly associated with lower ORs for poor IADL. In the final model (model 2), where the data were adjusted for social networks, social support, and social roles, as well as covariates, a significant inverse association of frequent participation with poor IADL was observed for only sports groups and senior citizens’ clubs among males: the ORs were 0.79 (95% CI, 0.64–0.97) in sports groups and 0.63 (95% CI, 0.41–0.97) in senior citizens’ clubs. In contrast, females had a significant inverse association between frequent participation and poor IADL for all types of SP: the ORs were 0.52 (95% CI, 0.27–0.99) in volunteer groups, 0.23 (95% CI, 0.14–0.39) in sports groups, 0.28 (95% CI, 0.18–0.45) in hobby groups, 0.30 (95% CI, 0.15–0.61) in senior citizens’ clubs, 0.15 (95% CI, 0.07–0.34) in neighborhood community associations, and 0.39 (95% CI, 0.16–0.94) in cultural groups.

**Table 3.** Odds ratios for poor instrumental activities of daily living associated with the type and frequency of social participation, stratified by gender (reference: non-participation of each group)

	Crude		Model 1^a^		Model 2^b^	
		
	OR	(95% CI)	OR	(95% CI)	OR	(95% CI)
**Males (*n* = 6935)**						
Volunteer groups						
Several times a year	0.52	(0.40–0.68)*	0.60	(0.45–0.79)*	0.75	(0.56–0.99)*
Several times a month	0.54	(0.41–0.71)*	0.69	(0.52–0.93)*	0.87	(0.65–1.17)
Once or more a week	0.54	(0.41–0.70)*	0.65	(0.49–0.87)*	0.82	(0.61–1.10)
Sports groups						
Several times a year	0.40	(0.30–0.54)*	0.53	(0.39–0.72)*	0.61	(0.45–0.84)*
Several times a month	0.44	(0.34–0.57)*	0.65	(0.49–0.85)*	0.78	(0.59–1.03)
Once or more a week	0.50	(0.42–0.60)*	0.68	(0.56–0.83)*	0.79	(0.64–0.97)*
Hobby groups						
Several times a year	0.51	(0.41–0.64)*	0.66	(0.53–0.84)*	0.82	(0.65–1.04)
Several times a month	0.41	(0.33–0.50)*	0.57	(0.46–0.71)*	0.70	(0.56–0.88)*
Once or more a week	0.46	(0.38–0.55)*	0.65	(0.53–0.79)*	0.80	(0.65–1.00)
Senior citizens’ clubs						
Several times a year	0.81	(0.61–1.05)	0.71	(0.53–0.95)*	0.87	(0.65–1.18)
Several times a month	0.72	(0.54–0.97)*	0.68	(0.50–0.94)*	0.85	(0.62–1.18)
Once or more a week	0.61	(0.41–0.90)*	0.53	(0.35–0.81)*	0.63	(0.41–0.97)*
Neighborhood community associations						
Several times a year	0.53	(0.46–0.62)*	0.68	(0.59–0.80)*	0.78	(0.67–0.92)*
Several times a month	0.41	(0.31–0.54)*	0.56	(0.42–0.74)*	0.70	(0.52–0.94)*
Once or more a week	0.58	(0.40–0.85)*	0.74	(0.50–1.10)	1.07	(0.71–1.60)
Cultural groups						
Several times a year	0.52	(0.40–0.69)*	0.66	(0.49–0.89)*	0.85	(0.63–1.15)
Several times a month	0.52	(0.37–0.71)*	0.75	(0.53–1.05)	0.95	(0.67–1.35)
Once or more a week	0.56	(0.37–0.85)*	0.71	(0.46–1.10)	0.84	(0.53–1.31)
**Females (*n* = 8021)**						
Volunteer groups						
Several times a year	0.25	(0.15–0.41)*	0.56	(0.32–0.99)*	0.97	(0.53–1.80)
Several times a month	0.19	(0.12–0.30)*	0.41	(0.23–0.73)*	0.74	(0.40–1.37)
Once or more a week	0.20	(0.13–0.33)*	0.30	(0.17–0.56)*	0.52	(0.27–0.99)*
Sports groups						
Several times a year	0.09	(0.03–0.29)*	0.31	(0.09–1.07)	0.63	(0.18–2.16)
Several times a month	0.04	(0.01–0.13)*	0.10	(0.03–0.32)*	0.15	(0.04–0.49)*
Once or more a week	0.06	(0.04–0.09)*	0.15	(0.09–0.25)*	0.23	(0.14–0.39)*
Hobby groups						
Several times a year	0.22	(0.14–0.34)*	0.34	(0.20–0.57)*	0.57	(0.33–0.99)*
Several times a month	0.11	(0.08–0.15)*	0.25	(0.17–0.36)*	0.37	(0.25–0.55)*
Once or more a week	0.07	(0.04–0.10)*	0.17	(0.11–0.27)*	0.28	(0.18–0.45)*
Senior citizens’ clubs						
Several times a year	0.80	(0.59–1.08)	0.53	(0.36–0.77)*	0.75	(0.50–1.12)
Several times a month	0.55	(0.41–0.75)*	0.35	(0.24–0.51)*	0.55	(0.37–0.81)*
Once or more a week	0.30	(0.17–0.55)*	0.20	(0.10–0.40)*	0.30	(0.15–0.61)*
Neighborhood community associations						
Several times a year	0.12	(0.09–0.16)*	0.33	(0.24–0.44)*	0.45	(0.33–0.62)*
Once or more a month	0.06	(0.03–0.12)*	0.11	(0.05–0.23)*	0.15	(0.07–0.34)*
Cultural groups						
Several times a year	0.10	(0.06–0.19)*	0.24	(0.12–0.48)*	0.36	(0.18–0.73)*
Several times a month	0.11	(0.06–0.19)*	0.19	(0.10–0.35)*	0.29	(0.15–0.57)*
Once or more a week	0.07	(0.03–0.15)*	0.20	(0.09–0.48)*	0.39	(0.16–0.94)*

CI, confidence interval; OR, odds ratio.

*P < 0.05.

^a^Adjusted for age, family structure, BMI, pensions, occupational status, the number of medications used, self-reported medical conditions, self-rated health, smoking, alcohol consumption, activities of daily living, depression, and cognitive function.

^b^In addition to the factors in Model 1, social network, social support, and social role were included.

### Additional analyses

To compensate for the effect of subjects with missing values, we performed a set of sub-analyses focusing on the 12 472 participants with perfect covariates. Overall, the results trended in the same direction (eTable 4). Regarding effect modification by gender, participation in all social groups except for senior citizen clubs showed significant interactions between SP and gender, demonstrating that females are more likely to benefit from active participation in social groups than males (eTable 5).

## DISCUSSION

The present study investigated the association between IADL and SP according to the type and frequency of participation in social groups in a cross-sectional study of 14 956 residents aged 65 years or over. Our results suggest that SP is significantly associated with a reduced prevalence of poor IADL, and this association increases with an increasing number of groups in which the subjects participated, regardless of gender. These associations are independent of the influence of demographics, health status, life-style habits, ADL, presence of depression, cognitive function, social networks, social support, and social roles. Prior studies have suggested that a higher level of social activity is associated with decreased risk of incident disability in IADL among community-dwelling older adults^8^ and that participation in a greater number of different organizations can reduce the onset of long-term care insurance certification in older people^9^; these findings are consistent with the results of the present study.

Several plausible mechanisms may explain the relationship between SP and IADL. First, as SP encourages individuals to practice activities of IADL (eg, using public transportation to attend social group meetings), elderly with SP may be prone to maintain independent IADL (the “use it or lose it” hypothesis). Second, participation in a wide range of social groups gives individuals an opportunity to access to various forms of material resources or health-relevant information.^33^ This may have an impact on behaviors relevant to health or protect people from stressful or other high-risk situations, resulting in independent IADL. Finally, SP may have a stress-buffering effect^33^ and psychological benefits. Prior studies have reported that SP of the elderly is associated with higher life satisfaction^34^ and higher self-esteem,^35^ and that positive psychological status, such as having a sense of purpose in life, may help community-dwelling elderly maintain IADL.^36^

In relation to the type and frequency of SP, our results have shown that significant associations vary by gender. For males, the beneficial effect of frequent participation was limited to sports groups and senior citizens’ clubs. In contrast, for females, frequent participation was significantly associated with a reduced prevalence of poor IADL in all types of social groups, and these significant associations were robust to adjustments for not only covariates including ADL, depression, and cognitive function, but also social networks, social support, and social roles. Kawachi et al have pointed out that frequent SP may bring about psychological distress for females and adversely impact their health (the role strain hypothesis).^37^ In contrast, Takagi et al found that SP had protective effects on depressive symptoms for females but not for males.^23^ Kavanagh et al also demonstrated that neighborhood-level political participation was favorable for women’s self-rated health, but not for men’s health.^38^ Our results showed that the inverse association between frequent SP and poor IADL was stronger for females than for males, which is inconsistent with Kawachi’s^37^ arguments but consistent with Takagi’s^23^ and Kavanagh’s^38^ reports. Because females have a wider range of emotional support sources^15^ and are more likely to make close friends from their networks,^39^ they may tend to receive positive benefits from SP; consequently, the association between females’ IADL and SP is stronger than that for males.

SP can not only have helpful effects on health but also unfavorable ones. Participation in social groups offers opportunities for the adverse aspect of social relationships, such as personal conflict, and the burden of obligation to the community.^33^ These harmful sides of SP lead to the potential for psychological stress. For males, our results showed no significant association of frequent participation in neighborhood community associations with IADL, but a significant association of comparatively infrequent participation with independent IADL. Takeuchi et al have pointed out that people often participate in neighborhood community associations obligatorily.^22^ Males who frequently participate in obligatory groups may feel pressured, negating any benefit of this type of SP on IADL. Taken together, these findings suggest that participation in non-mandatory social groups may bolster IADL for community-dwelling elderly, especially for males.

This study has some limitations. First, since this is a cross-sectional study, we cannot confirm causal relationships. Since poor IADL may restrain SP, longitudinal studies or intervention studies are needed to examine the effects of SP on IADL. Second, SP and IADL were assessed by self-report. Therefore, associations among the study variables may be overestimated due to a common method bias.^40^ Third, in the present study, SP was measured by the type and frequency of participation in social groups. Although this method is commonly used in Japanese studies on SP, it does not include assessment of SP continuity. Because the duration of SP is an important factor in measuring SP,^6^ our results should be confirmed using methods that include assessment of duration as well as the type and frequency of SP. Fourth, our results may be biased due to our exclusion of subjects who either could not supply required data or neglected to return the questionnaire. Proportions of individuals aged 75 years and older were greater among excluded individuals than among analyzed subjects (65.0% vs 39.8%). While we have no data about the non-responders, they may suffer from poor functional capacity or SP, which would have made their participation in this study impossible. It is our speculation that the population at highest risk for disability was left out of this study. This may have resulted in an underestimation of the association between SP and IADL. Finally, our study participants were the autonomous elderly living in urban communities in Nara, Japan. A review has pointed out that disability and neighborhood resources can influence SP among the elderly.^18^ Therefore, generalization of the findings to elderly citizens with disabilities or older people living in rural areas should be done with caution.

Despite these limitations, to our knowledge, the present study is the first to address the association between IADL and SP from the perspective of the number, type, and frequency of participation in social groups with regard to gender among the community-dwelling elderly. The present findings suggest that participation in a wider variety of social groups may influence independent IADL of community-dwelling elderly, and the beneficial effects of frequent participation on IADL may be more pronounced among elderly females than males. Our results imply that support for the non-disabled community-dwelling elderly that includes urging them to participate in social groups—taking their gender into consideration regarding the type of social groups they join and how often they participate in them—may be effective in promoting independent IADL and extending an active, healthy life.

## ONLINE ONLY MATERIALS

eTable 1. Tokyo Metropolitan Institute of Gerontology Index of Competence (TMIG-IC) for assessing higher-level functional capacity in older adults.

eTable 2. Distribution of the number of social groups, and the type and frequency of social participation (*n* = 14 956).

eTable 3. Characteristics of the analyzed subjects responding to the questionnaire (*n* = 14 956).

eTable 4. Odds ratios for poor instrumental activities of daily living among subjects with perfect covariates (*n* = 12 472).

eTable 5. Odds ratios for poor instrumental activities of daily living: gender and social participation interactions (*n* = 14 956).
