# Supplementary material for: Association Between Social Participation and Instrumental Activities of Daily Living Among Community-Dwelling Older Adults
Source: J Epidemiol. 2016 Oct 5;26(10):553–61. doi: 10.2188/jea.JE20150253 (PMC5037253; doi:10.2188/jea.JE20150253)
Supplement: eTable 1. [file je-26-553-s001.pdf]

eTable 1. Tokyo Metropolitan Institute of Gerontology Index of Competence for assessing higher-level functional capacity in older adults

Sub-scales		Response	
Instrumental Activities of Daily Living			
1	Can you use public transportation (bus or train) by yourself?	1. Yes	0. No
2	Are you able to shop for daily necessities?	1. Yes	0. No
3	Are you able to prepare meals by yourself?	1. Yes	0. No
4	Are you able to pay bills?	1. Yes	0. No
5	Can you handle your own banking?	1. Yes	0. No
Intellectual Activity			
6	Are you able to fill out forms for your pension?	1. Yes	0. No
7	Do you read newspapers?	1. Yes	0. No
8	Do you read books or magazines?	1. Yes	0. No
9	Are you interested in news stories or programs dealing with health?	1. Yes	0. No
Social Role			
10	Do you visit the homes of friends?	1. Yes	0. No
11	Are you sometimes called on for advice?	1. Yes	0. No
12	Are you able to visit sick friends?	1. Yes	0. No
13	Do you sometimes initiate conversations with young people?	1. Yes	0. No
