# Supplementary material for: Association Between Social Participation and Instrumental Activities of Daily Living Among Community-Dwelling Older Adults
Source: J Epidemiol. 2016 Oct 5;26(10):553–61. doi: 10.2188/jea.JE20150253 (PMC5037253; doi:10.2188/jea.JE20150253)
Supplement: eTable 2. [file je-26-553-s002.pdf]

eTable 2. Distribution of the number of social groups and the type and frequency of social participation (n=14,956)

	Males (n=6,935)		Females (n=8,021)		P^a
	n	%	n	%	
**Number of social groups**					
0	2,185	31.5	2,663	33.2	0.072
1	1,522	21.9	1,710	21.3	
2	1,251	18.0	1,352	16.9	
≥3	1,977	28.5	2,296	28.6	
**Type and frequency of social participation**					
Volunteer groups					
Non-participation	5,366	77.4	6,519	81.3	<0.001
Several times a year	553	8.0	425	5.3	
Several times a month	493	7.1	562	7.0	
Once a week	203	2.9	215	2.7	
Several times a week	210	3.0	228	2.8	
Four or more times a week	110	1.6	72	0.9	
Sports groups					
Non-participation	4,596	66.3	5,761	71.8	<0.001
Several times a year	490	7.1	165	2.1	
Several times a month	618	8.9	354	4.4	
Once a week	330	4.8	666	8.3	
Several times a week	602	8.7	756	9.4	
Four or more times a week	299	4.3	319	4.0	
Hobby groups					
Non-participation	4,039	58.2	4,533	56.5	<0.001
Several times a year	754	10.9	432	5.4	
Several times a month	1,049	15.1	1,468	18.3	
Once a week	477	6.9	726	9.1	
Several times a week	425	6.1	661	8.2	
Four or more times a week	191	2.8	201	2.5	
Senior citizens' clubs					
Non-participation	5,937	85.6	6,666	83.1	<0.001
Several times a year	407	5.9	473	5.9	
Several times a month	367	5.3	609	7.6	
Once a week	73	1.1	99	1.2	
Several times a week	122	1.8	135	1.7	
Four or more times a week	29	0.4	39	0.5	
Neighborhood community associations					
Non-participation	4,022	58.0	5,078	63.3	<0.001
Several times a year	2,127	30.7	2,337	29.1	
Several times a month	558	8.0	469	5.8	
Once a week	93	1.3	64	0.8	
Several times a week	83	1.2	53	0.7	
Four or more times a week	52	0.7	20	0.2	
Cultural groups					
Non-participation	5,865	84.6	6,298	78.5	<0.001
Several times a year	493	7.1	554	6.9	
Several times a month	370	5.3	666	8.3	
Once a week	127	1.8	283	3.5	
Several times a week	55	0.8	173	2.2	
Four or more times a week	25	0.4	47	0.6	

[a] Differences between males and females were analyzed using Fisher's exact test.
