# Supplementary material for: Association Between Social Participation and Instrumental Activities of Daily Living Among Community-Dwelling Older Adults
Source: J Epidemiol. 2016 Oct 5;26(10):553–61. doi: 10.2188/jea.JE20150253 (PMC5037253; doi:10.2188/jea.JE20150253)
Supplement: eTable 3. [file je-26-553-s003.pdf]

eTable 3. Characteristics of the analyzed subjects responding to the questionnaire (n=14,956)

	Males (n=6,935)		Females (n=8,021)		P^{a}
	n	%	n	%	
**Demographics**					
Age, years					
65-74	4,302	62.0	4,696	58.5	<0.001
≥75	2,633	38.0	3,325	41.5	
Family structure					
Living alone	492	7.1	1,422	17.7	
Living only with spouse	3,503	50.5	2,787	34.7	<0.001
Other	2,728	39.3	3,480	43.4	
Missing	212	3.1	332	4.1	
BMI					
Normal (18.5-<25.0 kg/m^2)	4,919	70.9	5,390	67.2	
Underweight (<18.5 kg/m^2)	355	5.1	895	11.2	<0.001
Overweight (≥25.0 kg/m^2)	1,385	20.0	1,366	17.0	
Missing	276	4.0	370	4.6	
Pensions					
National pension	781	11.3	3,613	45.0	
Employees' pension	4,748	68.5	2,934	36.6	
Mutual aid association pension	908	13.1	648	8.1	<0.001
Other	218	3.1	488	6.1	
Missing	280	4.0	338	4.2	
Occupational status (job with an income)					
Not engaged	4,904	70.7	6,715	83.7	
Engaged	1,898	27.4	1,041	13.0	<0.001
Missing	133	1.9	265	3.3	
**Health status**					
Number of medications used					
None	1,425	20.5	1,650	20.6	
1-2	1,846	26.6	2,197	27.4	
3-4	1,641	23.7	1,952	24.3	0.240
≥5	1,769	25.5	1,966	24.5	
Missing	254	3.7	256	3.2	
Number of illnesses under medical treatment					
None	1,276	18.4	1,449	18.1	
1	2,244	32.4	2,679	33.4	
2	1,617	23.3	1,900	23.7	0.011
3	947	13.7	957	11.9	
≥4	682	9.8	792	9.9	
Missing	169	2.4	244	3.0	
Self-rated health					
Very good	713	10.3	734	9.2	
Good	4,453	64.2	5,245	65.4	
Fair	993	14.3	1,208	15.1	0.006
Poor	372	5.4	449	5.6	
Missing	404	5.8	385	4.8	
**Life-style habits**					
Smoking status					
Never	1,701	24.5	7,013	87.4	
Former	3,809	54.9	420	5.2	<0.001

Current	1,137	16.4	260	3.2	<0.001
Missing	288	4.2	328	4.1	
Alcohol intake					
Nondrinkers	1,189	17.1	4,136	51.6	
Social drinkers	1,497	21.6	2,070	25.8	
Occasional drinkers	1,103	15.9	1,032	12.9	<0.001
Daily drinkers	2,856	41.2	500	6.2	
Missing	290	4.2	283	3.5	
**Physiological factor**					
Activities of daily living, Barthel-index					
Independent (score =100)	1,568	22.6	2,620	32.7	<0.001
Poor (score <100)	5,367	77.4	5,401	67.3	
**Psychological factors**					
Depression, Geriatric Depression Scale					
No depression (score <2)	4,964	71.6	5,433	67.7	
Depression (score ≥2)	1,641	23.7	2,272	28.3	<0.001
Missing	330	4.8	316	3.9	
Cognitive functioning, Cognitive Performance Scale					
Normal (score =0)	5,327	76.8	6,347	79.1	
Poor (score ≥1)	1,510	21.8	1,588	19.8	0.002
Missing	98	1.4	86	1.1	
**Social relationships**					
Social networks					
≥6	2,492	35.9	3,028	37.8	
3-5	1,425	20.5	1,918	23.9	
1-2	1,456	21.0	1,664	20.7	<0.001
None	1,202	17.3	989	12.3	
Missing	360	5.2	422	5.3	
Social support					
≥5	738	10.6	1,088	13.6	
3-4	2,122	30.6	2,675	33.3	
1-2	3,592	51.8	3,612	45.0	<0.001
None	220	3.2	354	4.4	
Missing	263	3.8	292	3.6	
Social roles, subscale of Tokyo Metropolitan Institute of Gerontology Index of Competence					
Independent (score =4)	3,100	44.7	4,465	55.7	<0.001
Poor (score <4)	3,835	55.3	3,556	44.3	

[a] Differences between males and females were analyzed using Fisher's exact test
