# Supplementary material for: Association Between Social Participation and Instrumental Activities of Daily Living Among Community-Dwelling Older Adults
Source: J Epidemiol. 2016 Oct 5;26(10):553–61. doi: 10.2188/jea.JE20150253 (PMC5037253; doi:10.2188/jea.JE20150253)
Supplement: eTable 4. [file je-26-553-s004.pdf]

eTable 4. Odds ratios for poor instrumental activities of daily living among subjects with perfect covariates (n=12,472)

	Males (n=5,883)		Females (n=6,589)	
	OR[a]	(95% CI)	OR[a]	(95% CI)
Number of social groups				
0	1.00		1.00	
1	0.76	(0.63 - 0.93) *	0.58	(0.43 - 0.78) *
2	0.73	(0.58 - 0.92) *	0.40	(0.26 - 0.59) *
≥3	0.68	(0.55 - 0.85) *	0.17	(0.10 - 0.28) *
p for trend		p=0.001		p<0.001
Volunteer groups				
Several times a year	0.81	(0.60 - 1.11)	0.77	(0.35 - 1.67)
Several times a month	0.99	(0.73 - 1.35)	0.79	(0.37 - 1.67)
Once or more a week	0.97	(0.71 - 1.32)	0.54	(0.26 - 1.13)
Sports groups				
Several times a year	0.64	(0.46 - 0.90) *	0.67	(0.19 - 2.41)
Several times a month	0.71	(0.52 - 0.97) *	0.19	(0.06 - 0.68) *
Once or more a week	0.79	(0.64 - 0.99) *	0.23	(0.12 - 0.41) *
Hobby groups				
Several times a year	0.84	(0.65 - 1.09)	0.58	(0.29 - 1.14)
Several times a month	0.78	(0.61 - 0.99) *	0.37	(0.24 - 0.58) *
Once or more a week	0.92	(0.73 - 1.16)	0.34	(0.20 - 0.57) *
Senior citizens' clubs				
Several times a year	0.90	(0.65 - 1.24)	0.78	(0.49 - 1.23)
Several times a month	0.96	(0.68 - 1.37)	0.57	(0.36 - 0.91) *
Once or more a week	0.64	(0.38 - 1.07) †	0.33	(0.13 - 0.81) *
Neighborhood community associations				
Several times a year	0.78	(0.65 - 0.92) *	0.50	(0.35 - 0.71) *
Several times a month	0.73	(0.54 - 1.00) *	0.15	(0.06 - 0.40) * b
Once or more a week	1.43	(0.92 - 2.22)		
Cultural groups				
Several times a year	0.89	(0.65 - 1.23)	0.29	(0.12 - 0.73) *
Several times a month	1.00	(0.69 - 1.45)	0.20	(0.09 - 0.48) *
Once or more a week	1.00	(0.62 - 1.62)	0.52	(0.21 - 1.31)

CI, confidence interval; OR, odds ratio.

† p<0.10

* p<0.05

[a] Adjusted for age, family structure, BMI, pensions, occupational status, the number of medications used, self-reported medical conditions, self-rated health, smoking, alcohol consumption, activities of daily living, depression, cognitive function, social network, social support, and social role
