# Supplementary material for: Association Between Social Participation and Instrumental Activities of Daily Living Among Community-Dwelling Older Adults
Source: J Epidemiol. 2016 Oct 5;26(10):553–61. doi: 10.2188/jea.JE20150253 (PMC5037253; doi:10.2188/jea.JE20150253)
Supplement: eTable 5. [file je-26-553-s005.pdf]

eTable 5. Odds ratios for poor instrumental activities of daily living: gender and social participation interactions (n=14,956)

	OR[a]	(95% CI)
Number of social groups		
1 (reference: zero)	0.51	(0.41 - 0.63) *
2 (reference: zero)	0.32	(0.23 - 0.43) *
≥3 (reference: zero)	0.10	(0.07 - 0.16) *
Male (reference: female)	1.65	(1.37 - 2.00) *
Participation number × gender	p<0.001	
Volunteer groups		
Several times a year (reference: non-participation)	0.59	(0.34 - 1.00) *
Several times a month (reference: non-participation)	0.49	(0.29 - 0.84) *
Once or more a week (reference: non-participation)	0.41	(0.24 - 0.70) *
Male (reference: female)	2.32	(1.97 - 2.73) *
Participation frequency × gender	p=0.002	
Sports groups		
Several times a year (reference: non-participation)	0.28	(0.08 - 0.90) *
Several times a month (reference: non-participation)	0.10	(0.03 - 0.31) *
Once or more a week (reference: non-participation)	0.16	(0.10 - 0.25) *
Male (reference: female)	2.09	(1.77 - 2.46) *
Participation frequency × gender	p<0.001	
Hobby groups		
Several times a year (reference: non-participation)	0.44	(0.27 - 0.71) *
Several times a month (reference: non-participation)	0.27	(0.19 - 0.39) *
Once or more a week (reference: non-participation)	0.19	(0.13 - 0.29) *
Male (reference: female)	1.92	(1.62 - 2.28) *
Participation frequency × gender	p<0.001	
Senior citizens' clubs		
Several times a year (reference: non-participation)	0.80	(0.56 - 1.12)
Several times a month (reference: non-participation)	0.59	(0.41 - 0.82) *
Once or more a week (reference: non-participation)	0.35	(0.19 - 0.66) *
Male (reference: female)	2.41	(2.04 - 2.84) *
Participation frequency × gender	p=0.474	
Neighborhood community associations		
Several times a year (reference: non-participation)	0.31	(0.23 - 0.41) *
Once or more a month (reference: non-participation)	0.14	(0.07 - 0.28) *
Male (reference: female)	2.00	(1.69 - 2.37) *
Participation frequency × gender	p<0.001	
Cultural groups		
Several times a year (reference: non-participation)	0.27	(0.14 - 0.53) *
Several times a month (reference: non-participation)	0.24	(0.13 - 0.43) *
Once or more a week (reference: non-participation)	0.21	(0.09 - 0.48) *
Male (reference: female)	2.20	(1.87 - 2.59) *
Participation frequency × gender	p<0.001	

CI, confidence interval; OR, odds ratio.

* $p<0.05$

[a]Adjusted for age, family structure, BMI, pensions, occupational status, the number of medications used, self-reported medical conditions, self-rated health, smoking, alcohol consumption, activities of daily living, depression, cognitive function, social network, social support, and social role
